# Safety of Laparoscopic Colorectal Surgery in a Low-Volume Setting: Review of Early and Late Outcome

**DOI:** 10.1155/2014/581523

**Published:** 2014-04-03

**Authors:** Robert C. Gandy, Christophe R. Berney

**Affiliations:** Department of Surgery, Bankstown-Lidcombe Hospital, University of New South Wales, Eldridge Road, Bankstown, NSW 2200, Australia

## Abstract

*Background*. There is increasing evidence suggesting that the laparoscopic technique is the treatment of choice for large bowel resection, including for malignancy. The purpose of the study was to assess whether general surgeons, with particular skills in advanced laparoscopy, can adequately provide safe laparoscopic colorectal resections in a low-volume setting. *Methods*. A retrospective review of prospectively collected case series of all laparoscopic colorectal resections performed under the care of a single general surgeon is presented. The primary endpoint was postoperative clinical outcome in terms of morbidity and mortality. Secondary endpoints were adequacy of surgical margins and number of lymph nodes harvested for colorectal cancer cases. *Results*. Seventy-three patients underwent 75 laparoscopic resections between March, 2003, and May, 2011. There was no elective mortality and the overall 30-day postoperative morbidity was 9.3%. Conversion and anastomotic leakage rates were both 1.3%, respectively. None of the malignant cases had positive margins and the median number of lymph nodes retrieved was 17. *Conclusions*. Our results support the view that general surgeons with advanced skills in minimally invasive surgery may safely perform laparoscopic colorectal resection in a low-volume setting in carefully selected patient cases.

## 1. Introduction

Since the first published series on large bowel resection using a minimally invasive approach in 1991 by Jacobs et al. [1], there has been convincing evidence in the literature to suggest that a laparoscopic approach should be the method of choice for colorectal resection. It is also suggested that oncological principles can be applied safely and adequately when compared to traditional open surgical approach [2]. However, some reservations still persist concerning the laparoscopic technique with regards to the management of rectal cancers where discrepancies between operators can be found in terms of disease-free survival and local recurrence [3, 4]. Although the assertion is relevant to low rectal cancers, its significance is independent of the method used to achieve tumour clearance and is therefore unrelated to the laparoscopic technique* per se* but more to the surgeon's familiarity with surgical anatomy and experience.

Despite the increasing trend in subspeciality training and fellowship numbers, a large proportion of colorectal resections will continue to be carried out by general surgeons outside tertiary referral hospitals. It is therefore widely accepted that open colorectal surgeries can be safely completed by generally trained surgeons with almost identical short- and long-term outcomes, bearing in mind that a large number of these cases are performed acutely for large bowel obstruction [5].

The progressive implementation of laparoscopy as a new tool in the repertoire of the specialist colorectal surgeons has demarcated them even further from mainstream general surgery. As a result, an increasing number of general practitioners are referring their patients to those specialist surgeons irrespective of their personal skills in minimally invasive surgery. In a vast country such as Australia, major colorectal resections are still part of general surgical training and will remain that way to allow for adequate general surgical services in remote rural areas and nontertiary centres where patients' transfers are often impossible. Furthermore, over the years a subset of general surgeons has developed advanced laparoscopic skills with a principal focus on gastrointestinal surgery. It is therefore presumed that some of these surgeons could also apply their skills to more advanced colorectal procedures in an attempt to offer similar benefit to their patients in terms of not only clinical outcome and length of stay but also overall hospital spending.

We hypothesise that most colorectal pathologies can be dealt with, laparoscopically, in a safe manner by fully trained general surgeons with advanced skills in minimally invasive surgery. We also believe that equivalent results can be obtained for benign or malignant conditions, excluding possibly low and middle rectal malignancies when compared to high-volume colorectal centres. The aim of this study is to retrospectively review the data of all patients who underwent laparoscopic colorectal resection under the care of a single general surgeon with broad experience in minimally invasive surgery but who performs less than 10 laparoscopic colorectal resections per year.

## 2. Material and Methods

Data was prospectively collected on all patients who were consented for laparoscopic colorectal surgery and stored in a dedicated clinical database. A minimally invasive approach was not offered to patients with locally advanced colorectal cancer or if there was an absolute contraindication to laparoscopy. Relative contraindications included emergency procedures, previous major abdominal surgery, or severe obesity (BMI > 35). Our local institutional review board approved the study. There were no conflicts of interests and the author did not receive any sponsorship for the design of this study. An initial evaluation of the collected information was presented at the 2011 Annual Scientific Congress of the Royal Australasian College of Surgeons [6].

Prior to conducting this study, the second author had exposure to the minimally invasive colorectal technique as an assistant or operator for one year and had also performed over 80 open colorectal resections himself. The surgical technique was standardized according to mentor and literature guidelines. In brief, all patients received a preoperative single dose of intravenous 2 g ceftriaxone with 500 mg metronidazole. Sequential calf compressors and subcutaneous 20 mg enoxaparin were used for deep venous thrombosis prophylaxis. An indwelling catheter (IDC) was routinely inserted as well as placement of a nasogastric tube, which was systematically removed prior to extubation. A three- to five-port technique was employed, with a carbon dioxide insufflation pressure of 12 mmHg. An ultrasonic scalpel (Harmonic ACE Curved Shears, Ethicon Endo-Surgery (EES), Cincinnati, OH, USA) was used preferentially for tissue dissection and vascular pedicles were divided using an articulated ATW45 linear stapler (EES). The bowel was exteriorised through a small tailored transverse incision, previously protected with an Alexis wound protector (Applied Medical, Stafford, QLD, Australia), for resection and extracorporeal anastomosis. Following colonic resections, a mechanical end-to-end anastomosis was preferentially fashioned using a double-stapling technique with a size 75 mm linear stapler device and one reload (TLC75, EES). Rectal anastomoses were systematically achieved with size 29 mm circular staplers (EES). In instances of low or middle rectal cancers a total mesorectal excision was routinely performed. Conversion from laparoscopic to open bowel resection was directed by the surgeon in cases of technical difficulties or if patient safety was compromised. Postoperative care, including early feeding, cessation of patient-controlled analgesia (PCA), and removal of IDC, was adapted to each patient but preferentially following a “fast-track” colon surgery program [7].

Patients were reviewed 2 and 6 weeks postdischarge. Colonoscopy was performed 3 months postsurgery if not completed preoperatively. For all bowel cancer patients a clinical review with FBC, EUC, LFTs, and CEA levels was performed every 6 months for the first 2 years and then yearly thereafter. A yearly computerised tomography (CT) of the abdomen and pelvis, with or without chest CT, was organised for up to 5 years. Finally, colonoscopy was performed yearly for the first 2 years and at 5-year intervals thereafter if initially unremarkable. Patients with rectal cancer had alternating rigid sigmoidoscopy and colonoscopy every 6 months for 2 years and then a yearly colonoscopy up to 5 years postoperatively.

This paper is chiefly descriptive and only represents one surgeon's operative data; hence, no control group is available. In addition, the relatively small number of patients operated on for colorectal malignancy made it superfluous to use Kaplan-Meier curves to estimate the distribution of disease-free and overall survival.

## 3. Results

From March, 2003, to May, 2011, a total of 110 patients underwent 113 colorectal resections under the care of one surgeon (Christophe R. Berney). Out of thirty-seven cases scheduled as open procedures, 31 of them (83.8%) took place during the first 4 years of this review and were performed as an emergency in 17 cases (54.8%), whereas the remaining 6 open resections performed during the second half of this study were urgent on 5 occasions (83.5%). During this 8-year period, 73 selected patients underwent 75 laparoscopic (66.4% of 113 procedures) colorectal resections. There were 37 male and 36 female patients with a mean age of 65 years old (range: 24–90 years). Baseline demographics and clinical characteristics are summarised in Table 1. The procedure was elective in 88% of the cases and one patient had to be converted into laparotomy (1.3%), due to haemodynamic instability.

The most common indication for surgery was the presence of a malignancy (57.3%, 43/75 cases), while 16% had a large sessile polyp not amenable to endoscopic resection (12/75). Smaller numbers were performed for diverticular disease of the sigmoid colon (9.3%, 7/75) and inflammatory bowel disease (6.7%, 5/75), as shown on Table 2. Type and number of colorectal resections, including other associated surgical procedures, are summarised in Table 3. The majority of the procedures were laparoscopic right hemicolectomy (28 cases) and high anterior resection (16 cases) and accounted for 58.7% of the 75 cases. It should be noted that in twenty-one instances additional pathologies were treated synchronously and completed laparoscopically: the most common procedure was a laparoscopic cholecystectomy (8/21 cases).

The median length of stay (LOS) for elective cases was 5 days and 10 days for emergencies. The overall 30-day morbidity and mortality rates were 9.3% (7/75) and 2.7% (2/75), respectively. When analysing elective and emergency procedures separately, we did not encounter any mortality among the 66 elective bowel resections, but two (22.2%) occurred out of the 9 urgent laparoscopic cases (Table 4). The first patient was a 79-year-old male who underwent an emergency anterior resection for an obstructing and locally infiltrating colon cancer. Unfortunately, he subsequently developed aspiration pneumonia and, despite admission to ICU for respiratory failure and tracheal reintubation, died of cardiorespiratory arrest on the 17th postoperative day. The second one, 82-year-old man with ischaemic heart disease and dementia, was initially admitted on emergency to our institution for a left hip fracture. During this admission he developed recurrent episodes of sigmoid volvulus and finally required sigmoid colectomy. His postoperative recovery was initially uneventful, but he succumbed unexpectedly two weeks later from decompensated cardiac failure.

Two patients (2.7%) had delayed complications and were treated conservatively. One of them, a 65-year-old woman with previous history of hysterectomy, developed an anastomotic leak (1.3%) six weeks following laparoscopic low anterior resection for stage II rectal cancer (Table 4). Her main clinical symptom was occasional passage of vaginal flatus. The rectovaginal fistula spontaneously closed within several weeks with conservative management. The second one, an 84-year-old female patient who underwent laparoscopic right hemicolectomy for locally advanced caecal cancer (T4N3 M0), was discharged home on the 7th day postoperatively. She was subsequently treated with anticoagulation for pulmonary embolism that occurred 4 weeks later.

When only looking at the 43 operations completed for primary colorectal cancer, there was a slightly lower male/female ratio. The procedure was performed electively in 86% (37/43) of those cases with a median LOS of 6 days. The median number of harvested lymph nodes was 18 (range: 5–58) and there was no positive resection margin on histopathology. Twenty-four of those cases were categorized as stage I or II colorectal cancers on final histopathological review as per TNM classification, 13 were stage III, and the remaining 6 patients had already distant metastases at the time of surgery (stage IV). After a median followup of 48 months, one patient died two years postsurgery from an unrelated metastatic oesophageal cancer, one patient was diagnosed 10 months postoperatively with metastatic bladder cancer but is still cancer-free in regard to his previous colonic resection, and one patient developed two liver metastases and one lung metastasis 2 and 4 years, respectively, following initial laparoscopic high anterior resection for stage II rectosigmoid cancer. All three lesions were successfully excised and the patient is currently cancer-free after a followup of 60 months. Finally, two patients who had undergone surgery for advanced colorectal carcinoma developed peritoneal recurrence. This occurred three years following a laparoscopic right hemicolectomy for T4N2M0 ascending colon cancer in the first one, and in the second case the diagnosis was made two and half years after he underwent a laparoscopic right hemicolectomy for T3N2M0 caecal cancer. Regrettably, this last patient who was only 38 years of age at time of surgery categorically refused adjuvant chemotherapy.

## 4. Discussion

The biggest challenge faced by the general surgeon is the acquisition and mastering of the advanced laparoscopic skills that will allow him to deal with more complex procedures. The availability of specific equipment, such as laparoscopic sealing and stapling devices, and the surgeon's familiarity with their use are also both essential to achieve excellent results in a wider range of pathologies that include colorectal resections. High conversion rates of 17–29% were observed in early-randomised controlled trials for bowel cancer [8–10]. Interestingly, these studies included only accredited surgeons who had performed at least 20 laparoscopically assisted cases. A recent meta-analysis reviewing all clinical studies published between January, 1994, and January, 2005, on the efficacy and safety of laparoscopic versus open approaches for colorectal disease confirmed slightly better conversion rates estimated at 14.8% for malignancy, 11.1% for diverticular disease, and 8.1% for inflammatory bowel disease patients [11]. In our series, only one case (1.3%) was converted to open surgery. We realise that selection bias must have played a significant role in this study as the laparoscopic approach was initially offered to carefully chosen patients with limited BMI index. This may in part explain our surprisingly low conversion rate.

It is interesting to see that when separating elective and emergency surgery, the 30-day morbidity and mortality rates were considerably different (Table 4). These results can be easily explained by several factors. First, emergency surgery carries a higher risk of complications and/or death. Second, the 9 patients operated on emergency were on average 13 years older than those treated electively. Three, overall the elective surgery group, had a significantly better anaesthetic risk as per ASA grade (data not shown). This also explains why the median LOS for elective cases was half the length of emergency operations.

Despite early concerns regarding the use of laparoscopy for treatment of colonic and rectal cancers, good evidence now exists to support its equivalence to the open approach [8–10, 12]. Similarly, a recent analysis from the COST study group confirmed that the type of surgery chosen for treatment of curable colon cancer did not influence 5-year survival [13]. Although not being part of our primary or secondary endpoints we will briefly discuss the cancer-free survival (CFS) figures of our 36 patients treated for 37 stages I–III colorectal cancers (one patient developed a metachronous transverse colon cancer 14 months after laparoscopic low anterior resection for stage II rectal cancer). We recognise that these results ought to be interpreted with caution in view of the small numbers involved and limited followup. Nonetheless, our findings suggest that a skilled laparoscopic surgeon can perform safe and adequate oncological colorectal resection with acceptable CFS, with a median followup of 48 months. Although we have included a very small number of rectal malignancies in this series, there were no local recurrences following any of the eight laparoscopic anterior resections performed for carcinoma. Finally, only one of those patients developed a delayed rectovaginal fistula that spontaneously healed with conservative management. This low incidence of anastomotic leak may be also partly explained by a good patients' selection and the fact that the majority of them (16 out of 23) who required laparoscopic anterior resection had an estimated preoperative ASA grade of 1 or 2 [14].

There has been much debate in the literature and no real consensus about the minimum number of lymph nodes necessary to adequately stage colorectal cancers. General recommendation today is to harvest at least 12–15 lymph nodes to accurately predict regional node negativity [15]. In our case series, the median number of lymph nodes harvested for malignancies was 18 and compares favourably to previously published studies [11]. The overall median LOS of our patients operated on for colorectal malignancy was 6 days. These results are similar to an overall reported mean LOS of 7.8 days in a recent review [11].

An increasing number of general surgeons are already using these minimally invasive tools more broadly and more regularly in their practice; we firmly believe that some of them would be able to adapt to laparoscopic colorectal resections in a more efficient and safe manner, irrespective of their personal caseload. Indeed, previous trials repeatedly returned results reflecting little or no detectable caseload effect for surgeons managing colorectal cancer [16–18]. Laparoscopic colorectal surgery has a steep learning curve [19, 20] but it can be safely taught to surgical fellows with no prior experience with laparoscopic colorectal resection [21]. Our results support the fact that this learning curve can be reduced for those general surgeons who already possess advanced laparoscopic skills. Consequently, we might also extrapolate that laparoscopy could also be offered in emergency situations and in a carefully selected patient group, such as in our study where this approach was offered on nine occasions (12%). Having said that we acknowledge that only surgeons who can maintain equivalent or better results, as compared to their previous open bowel resection experience should advocate this alternative laparoscopic approach. This is even more relevant to cancer surgery. We therefore recommend surgeons to perform long-term longitudinal followup of their patients and to maintain clinical record in a dedicated database for further review.

Laparoscopic proctectomy for rectal cancer can be particularly challenging for both the general and maybe to a lesser degree specialist colorectal surgeons. In those cases the surgical technique remains the most important treatment modality in terms of cure, staging, risk of local recurrence, and prognosis. This is particularly true for middle and low rectal cancers where a meticulous total mesorectal excision is of paramount importance [22]. In those particular cases high-volume centres have produced better results than surgeons with limited caseloads [23–25] and also these findings have been challenged by others [26, 27]. Although we agree that specialist surgeons with wide expertise in pelvic dissection are likely to produce better clinical outcomes, we trust that the quality of the surgeons' laparoscopic skill set could also influence those results, irrespective of their caseload, and should not be ignored. Interestingly, two recent studies confirmed tha,t in rectal excision for cancer, laparoscopic approach had similar long-term local control and cancer-free survival than open surgery [28, 29].

Finally, out of the 21 additional pathologies treated synchronously during bowel resections the most common procedure was a laparoscopic cholecystectomy for cholelithiasis on eight occasions. Although this number might appear relatively elevated, the indication was motivated by the presence of associated bowel cancer [30], recurrent episodes of biliary colic, or, on one occasion, after total colectomy for ulcerative colitis [31].

## 5. Conclusion

Qualified general surgeons with advanced laparoscopic skills are likely to perform colorectal resections safely and with adequate short- and long-term clinical outcome in selected patients. In order to achieve broader acceptance from our colleagues these results ought to be replicable and comparable to those of specialist colorectal surgeons, ideally in well-designed prospective cohort studies. In the meantime, patients with middle or low rectal cancers should be preferably referred to high-volume centres.

## Figures and Tables

**Table 1:** Summary of baseline demographics and clinical characteristics of 73 patients undergoing 75 laparoscopic large bowel or rectal resections.

Mean age (years) (range)	65 (24–90)
Male/female ratio	37/36
ASA classification (73 patients)	
I	9
II	40
III	19
IV	5
Elective	66
Emergency	9
Intraoperative complication	0
Conversion to open surgery (%)	1 (1.3)
Median length of stay	
Elective (range)	5 (3–15)
Emergency (range)	10 (5–35)

**Table 2:** Indications for surgery.

Colorectal cancer	43
(i) Caecum	10
(ii) Ascending colon	7
(iii) Transverse colon/hepatic flexure	5
(iv) Descending colon/splenic flexure	4
(v) Sigmoid colon	8
(vi) Rectosigmoid junction	3
(vii) Rectum	5
(viii) Pelvic metastatic	1
Colorectal polyp (sessile)	12
(i) Caecum	4
(ii) Hepatic flexure	2
(iii) Sigmoid colon	3
(iv) Rectum	3
Carcinoid	3
(i) Appendix	2
(ii) Terminal ileum	1
Lymphoma	1
Benign bowel disorders	15
(i) Crohn's disease	4
(ii) Ulcerative colitis	1
(iii) Ischaemic colitis	2
(iv) Diverticulitis/diverticular abscess	7
(v) Calcified mesenteric cyst	1
Sigmoid volvulus (recurrent)	1
Total	**75**

**Table 3:** Summary of 75 laparoscopic colorectal resections and 21 associated procedures.

Ileocaecal resection	5
Right hemicolectomy	28
Extended right hemicolectomy	2
Left hemicolectomy	4
Sigmoid colectomy	7
Anterior resection	23
(i) High	16
(ii) Low	6
(iii) Ultra low	1
Partial colectomy	4
Total colectomy	1
Hartmann's procedure	1
Total	**75**

Cholecystectomy	8
Small bowel resection	1
Bilateral oophorectomy	2
Unilateral oophorectomy	1
Liver biopsy	2
Loop ileostomy	2
Closure colovesical fistula	2
Closure enterocutaneous fistula	1
Hysterectomy	1
Mesh repair ventral hernia	1
Total	**21**

**Table 4:** Summary of morbidity and mortality rates following 75 laparoscopic large bowel or rectal resections.

Elective (%)	66 (88)
Mean age (years) (range)	63 (24–89)
Male/female ratio	34/32
30-day mortality (%)	0 (0)
30-day morbidity (%)	4 (6)
Superficial wound infection	2
Prolonged ileus	1
Postoperative atrial fibrillation	1
Emergency (%)	9 (12)
Mean age (years) (range)	76 (47–90)
Male/female ratio	4/5
30-day mortality (%)	2 (22.2)
30-day morbidity (%)	3 (33.3)
Superficial wound infection	1
Pleural effusion	1
Urinary retention	1
Delayed (>30 days) morbidity (%)	2 (2.7)
Pulmonary embolism	1
Clinical rectovaginal fistula	1
